# Sortilin Expression Is Essential for Pro-Nerve Growth Factor-Induced Apoptosis of Rat Vascular Smooth Muscle Cells

**DOI:** 10.1371/journal.pone.0084969

**Published:** 2014-01-03

**Authors:** Luisa Campagnolo, Gaetana Costanza, Arianna Francesconi, Gaetano Arcuri, Ilana Moscatelli, Augusto Orlandi

**Affiliations:** 1 Department of Biomedicine and Prevention, Anatomic Pathology Institute, University of Rome “Tor Vergata”, Rome, Italy; 2 Department of Molecular Medicine and Gene Therapy, Lund Stem Cell Center, University of Lund, Lund, Sweden; University of Bristol, United Kingdom

## Abstract

**Background:**

Sortilin, a member of the Vps10p-domain receptor family, has been demonstrated a key regulator in mediating cellular response to pro-neurotrophins. In the present study, we investigated the role of sortilin in the apoptotic pathway of vascular smooth muscle cells.

**Methods and Principal Findings:**

Immunohistochemistry revealed that sortilin was barely detectable in human and rat normal young vessels, while its expression was increased in human fibroatheromatous plaques. Sortilin immunodetection was also marked in the neointima of the rat aorta fifteen days after ballooning. *In vitro*, rat aortic intimal cells expressed higher sortilin levels than normal media SMCs; sortilin was distributed in the cytoplasm and in correspondence of the cell membrane. After 48 h, pro-nerve growth factor (proNGF) induced the strong dose-dependent increase of intimal cell apoptosis and the accumulation of sortilin protein. ProNGF was a more potent apoptotic inducer than equimolar or even higher concentration of NGF, whereas brain derived neutrotrophic factor was ineffective. Targeted interfering RNA-mediated sortilin reduction counteracted proNGF-induced apoptosis without affecting p75^NTR^ expression. ProNGF-induced apoptosis was associated to NF-κB down-regulation and bax increase. Inhibition of NF-κB activity increased intimal cell apoptosis that did not further increase with the addition of proNGF.

**Conclusions:**

Our results indicate that sortilin expression characterizes human atheromatous lesions and rat aortic post-injury neointima, and suggest that sortilin represents an important regulator of proNGF-induced SMC apoptosis and arterial remodeling.

## Introduction

Smooth muscle cell (SMC) accumulation within the intima characterizes human atheromatous plaque and restenosis following angioplasty or stenting procedures [1], [2]. SMC accumulation results from the imbalance between proliferative [1], [2] and pro-apoptotic signals [3], [4]. SMC apoptosis is extremely rare in normal adult vessels and becomes evident in human atherosclerotic plaque and restenosis [4], [5]. Experimental post-injury intimal thickening provides useful information concerning SMC apoptotic behaviour [3], [6]. Immediately after damage by ballooning, normal media SMC apoptosis rapidly occurs [7]. Successively, apoptotic death contributes to counteract excessive neointimal SMC hyperplasia [3]. In fact, despite the prolonged proliferative activity, the total number of intimal SMCs remains constant [8]. Intimal SMCs display phenotypic changes [9], [10] with the modulation of antigenic and receptor status that influences SMCs response to microenvironmental changes [11], [12]. In addition, intimal SMCs display an increased apoptotic susceptibility compared to normal media SMCs [13]. Growth factors and cytokines regulate SMC dedifferentiation toward a synthetic phenotype [14], [15]. Neurotrophins, a family of polypeptide growth factors that includes nerve growth factor (NGF), brain derived neurotrophic factor (BDNF), neurotrophins 3 (NT3) and 4/5 (NT4/5) [16], have been demonstrated to be expressed in SMCs. Among neurotrophins, NGF has been reported to stimulate apoptosis in different cell types, including vascular SMCs [17]. Neurotrophins are synthesized as precursor forms (proneurotrophins), which dimerize after translation [18]. Proneurotrophins can be either secreted as such [19] or cleaved to generate mature neurotrophins [20]. Proneurotrophins *per se* have been recently demonstrated to exert biological functions [21]. In particular, proNGF has been shown to induce cell apoptosis through its binding to the p75 neurotrophin receptor (p75^NTR^) [20], [22]. In order to mediate such effect, p75^NTR^ associates with sortilin, a member of the Vps10p-domain receptor family, expressed in embryonic as well as in adult tissues [22], [23]. p75^NTR^ has been detected in human atherosclerotic plaques and in rat aortic post-injury intimal thickening [24] and is implicated in SMC apoptosis [17]. Stimulation of p75^NTR^ activates the transcription factor Nuclear Factor-kappaB (NF-κB), which is involved in a variety of physiological functions, including cell survival and death [25], [26]. In particular, NF-κB plays a relevant role in vascular SMC apoptosis according to cell culture condition and phenotype [27]. Although functionally associated with p75^NTR^, little information is available about sortilin distribution and its specific contribution to vascular remodeling, and in particular to SMC apoptosis. Here we described the expression of sortilin in human atherosclerotic lesions and rat post-injury aortic neointimal cells *in vivo* and *in vitro*. We also demonstrated that sortilin intracellular redistribution and accumulation appear essential for proNGF-induced apoptosis of vascular SMCs, likely through the modulation of NF-κB activity. These findings support a relevant role of sortilin in post-injury arterial remodeling.

## Materials and Methods

### Materials

Cell culture reagents were obtained from Invitrogen (San Diego, CA, USA), unless otherwise specified. Fetal bovine serum (FBS) was from Biological Industries (Haemek, Israel) and oligofectamine and G418 from Invitrogen. NGF and BDNF from Peprothec (Rocky Hill, NJ). ProNGF from Scil Proteins GmbH (Halle, Germany) and ammonium pyrrolidinedithiocarbamate (PDTC) from Sigma-Aldrich (St. Louis, MO USA). Antibodies used in this study were the following: mouse anti-α-smooth muscle actin (α-SMA, 1∶500; Dako, Dakopatts, Denmark), anti-smooth muscle myosin (1∶400; NeoMarkers, Fremont, CA USA), anti-CD68 (Dako, 1∶250), anti-α-tubulin and anti-Chromosome Region Maintenance 1 (CRM1, 1∶1000; Sigma-Aldrich), rabbit anti-sortilin (1∶200, Abcam, Cambridge, UK; Chemicon Intern, Temecula, CA USA), anti-proNGF (Sigma-Aldrich), anti-bax protein (1∶200), anti-NF-κB p65 (1∶200), anti-p50 (1∶100), anti-IκB-α (1∶50), goat anti-p75^NTR^ (1∶200), anti-bcl-2 (1∶100, Santa Cruz Biotechnology, CA, USA) and anti-hypoxanthine-guanine phosphoribosyltransferase (HPRT; Abcam). Fluorocrome conjugated secondary antibodies were purchased from Jackson (Suffork, UK) and Invitrogen. Horseradish peroxidase-conjugated secondary antibodies were from Nordic (Tilburg, The Netherlands).

### Vascular Tissues

Paraffin sections of formalin-fixed tissue samples of human grossly normal young (n = 7, median age 21.5±1.9 yrs) and old donor aortas (n = 7, median age 67±3 yrs), and atherosclerotic aortic and carotid vessels (n = 6, median age 63.1±2 yrs) were obtained from block archive of Anatomic Pathology, Tor Vergata University of Rome, Italy. Approval for the use of human derived tissues have been requested and obtained by the Internal Ethical Committee of this University. Written informed consent was obtained for the original human work that produced the tissue samples. Paraffin sections from formalin-fixed rat aortic tissue of two month-old Wistar uninjured rats (n = *7*), three (n = 4), seven (n = 5), fifteen (n = *6*) and forty-five (n = *6*) days after ballooning [28] used. All procedures were performed in triplicate. Animal protocols were approved by the Institutional Animal Care and Use Committee of the University of Rome Tor Vergata (Interdepartmental service center, Animal technology station), and comply with European rules (116/92).

### Vascular Smooth Muscle Cell Populations

For the *in vitro* studies, rat aortic intimal cells obtained fifteen days after ballooning (IT cells) and uninjured normal media SMCs (mSMCs) were isolated by enzymatic digestion, as previously reported [12]. The myocitic nature of cells in primary cultures was confirmed by anti-α-SMA and anti-SM myosin immunostaining [11]. For protein extraction, cells were seeded at a density of 2.5×10^3^ cells/cm^2^ and collected in lysis buffer after 3 and 6 days for sparse and confluent culture conditions respectively. For neurotrophin and proNGF stimulation, 80% confluent cultures were serum-starvated for 24 hours before treatment.

### Immunohistochemistry and Immunofluorescence

Immunostaining for sortilin, p75^NTR^ and α-SMA of vascular tissues was performed on serial paraffin-embedded sections and the percentage of positive cells calculated [29]. For immunofluorescence [30], 4% paraformaldehyde-fixed cells were stained with anti-sortilin (1∶100) and anti-p75^NTR^ (1∶100) antibodies, followed by TRITC-conjugated goat anti-rabbit and Alexa fluor-conjugated donkey anti-goat antibodies.

### Apoptosis Assay

Apoptotic DNA double strand breaks were measured by terminal deoxynucleotidyl transferase mediated fluorescin-dUTP nick end labeling (TUNEL) assay, Hoechst staining and cell cytometry, and results reported as percentages of nuclei showing apoptotic features or of subG1 fraction, respectively [28]. In order to better identify and quantify apoptosis-associated DNA fragmentation, DNA was extracted, and ligation-mediated PCR was performed [28].

### Protein and RNA Studies

Total protein and mRNA extraction, quantification and blotting were performed as previously reported [28]. RT-PCR and real time PCR were performed using the following primer sequences: Sortilin, forward 5′-GGGGACCAAACAACATCATC-3′ and reverse 5′-AAGGGCTCATGACCACAGTC-3′; p75^NTR^, forward 5′- TCGCCAGTGGACACTAACAG-3′ and reverse 5′-AGGAGAGAACCTGCTGGTGA-3′; TrkA, forward 5′-GAGTCTGATGCGAGCCCTAC-3′ and reverse 5′-AGACGTTCTAGTCCTGTG-3′; TrkB, forward 5′-CACAGAGGCGAAGAGGAAAC-3′ and reverse 5′-GGATGCAGGGATGATGTTCT-3′; TrkC, forward 5′-TGATCCCAAATTTGCTCCCT-3′ and reverse 5′-CTCGGTGGTGAATTTCCTGT-3′; hypoxanthine-guanine phosphoribosyl-transferase (HPRT), forward 5′-GACCGTCGACCGTACTTGTT-3′ and reverse 5′-CCTACAGGCTCATAGTGCAAA-3′. EMSA analysis and supershift assays were performed as reported [28], using an NF-κB-specific oligonucleotide (Invitrogen). The sequence was as follows: 5′-AGTTGAGGGGACTTTCCCAGGC-3′. NF-κB antibodies used for supershift EMSA were anti-p50 and anti-p65 (Santa Cruz).

### Sortilin SiRNA Design

Double-stranded siRNA against sortilin and sortilin scramble sequences were designed using the Invitrogen siRNA wizard software. Sequences were then cloned into the psiRNAhH1neoG2 plasmid (Invitrogen), using BbsI restriction sites. The following target sequences were used: sortilin (GenBank™: accession number AF_019109) 5′-ACCTCG CTGATAAGGATACAACAAGATCAAGAGTCTTGTTGTATCCTTATCAGCTT-3′; 5′-CAAAAAGCTGATAAGGATACAACAAGACTCTTGATCTTGTTGTATCCTTATCAGCG-3′; scramble 5′-ACCTCGGAAGCAGACAACAGATAATTTCAAGAGAATTATCTG TTGTCTGCTTCCTT-3′; 5′-CAAAAAGGAAGCAGACAACAGATAATTCTCTTGAAA TTATCTGTTGTCTGCCTTCCG-3′. Plasmids were sequenced to verify that the inserts had the correct reading frames and sequences. Knock-down efficiency was evaluated after 24 h by blot and immunofluorescence analysis.

### Statistical Analysis

Results were expressed as mean ± SEM of three different experiments. For comparison between two groups, Student’s *t* test was used. Statistical significance was determined at a value of *p*<0.05.

## Results

### Sortilin Expression Characterizes Human Pathological Vessels

**Figure 1:**
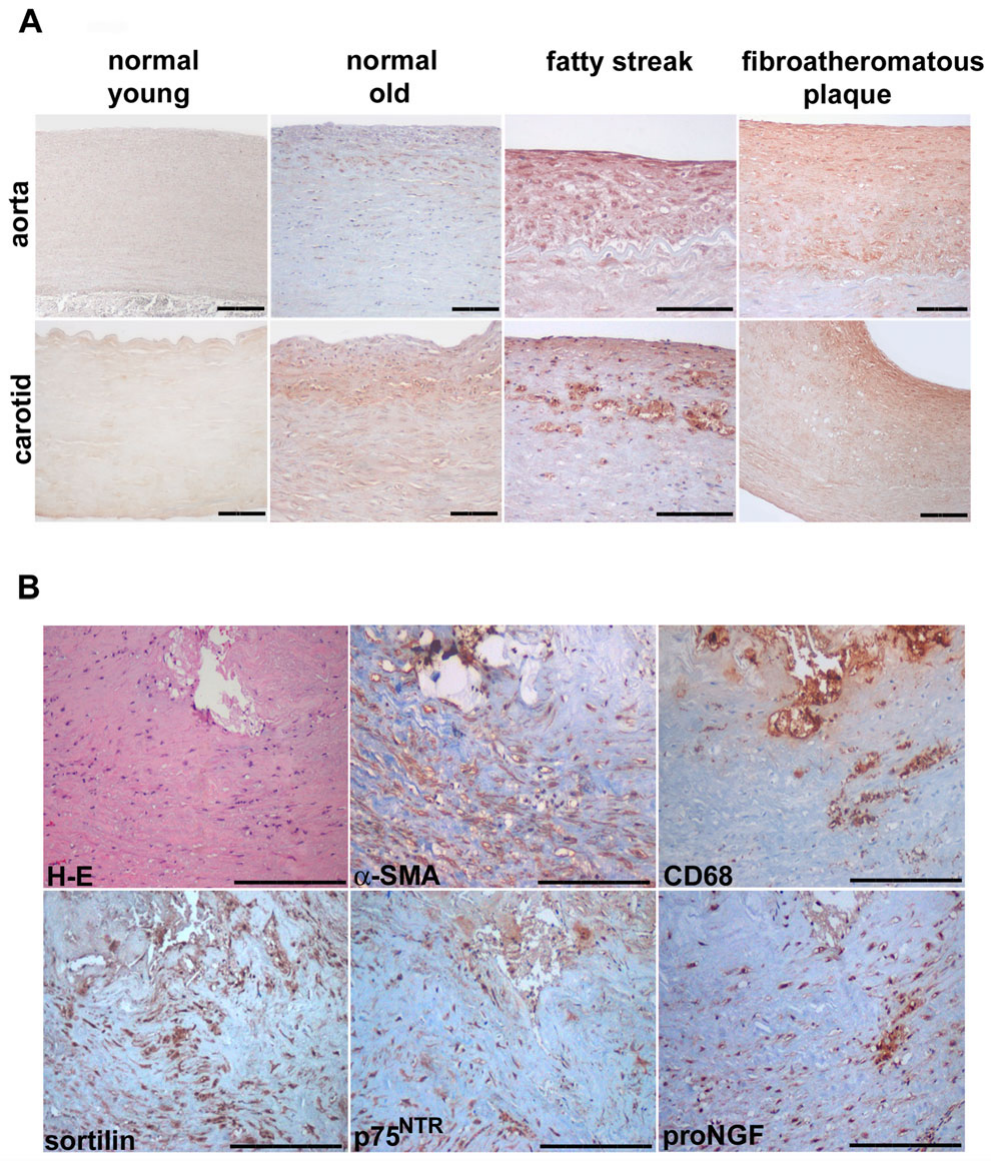
Sortilin immunostaining of grossly normal human young and old and atherosclerotic aorta and carotid vessels. Normal young vessels (A) do not display appreciable sortilin immunodetection; the latter is observed in old vessel intimal thickening and fatty streak and, more markedly, in fibroatheromatous plaque. Representative images (B) of serial sections of human fibroatheromatous plaque stained with Haematoxylin-Eosin (H-E), α-smooth muscle actin (α-SMA), CD68, sortilin, p75^NTR^ and proNGF. Diaminobenzidine as chromogen, Haematoxylin counterstaining. Scale bar = 50 µm.

In human aged donor aortas and carotids, a diffuse intimal thickening was clearly evident, and much more greater (330±11 and 117±19 µm) compared to young donors vessels (31±6 and 11±2 µm), according to previously reported findings [31]. Rare CD68^+^ cells were observed the diffuse intimal thickening, as reported [31], and their number increased in fatty streaks. As reported in Figure 1A, in grossly normal young aortas and carotids, sortilin immunostaining was faint or absent. Conversely, sortilin was similarly immunodetected in diffuse intimal thickening of grossly normal old vessels and fatty streaks, and appeared markedly increased in fibroatheromatous plaques. Intimal elongated myocitic and rounded foamy cells appeared both sortilin immunopositive, whereas the endothelium and underlying tunica media cells were almost negative. The vast majority of intimal and almost the totality of medial aortic cells showed a positive immunostaining for the myocitic markers. Immunostaining of serial sections of human atheromatous plaques (Fig. 1B) documented the increased sortilin expression in both α-SMA and CD68 positive intimal areas.

### Sortilin Expression is Increased in Rat Aortic Intimal Thickening Early after Injury

**Figure 2:**
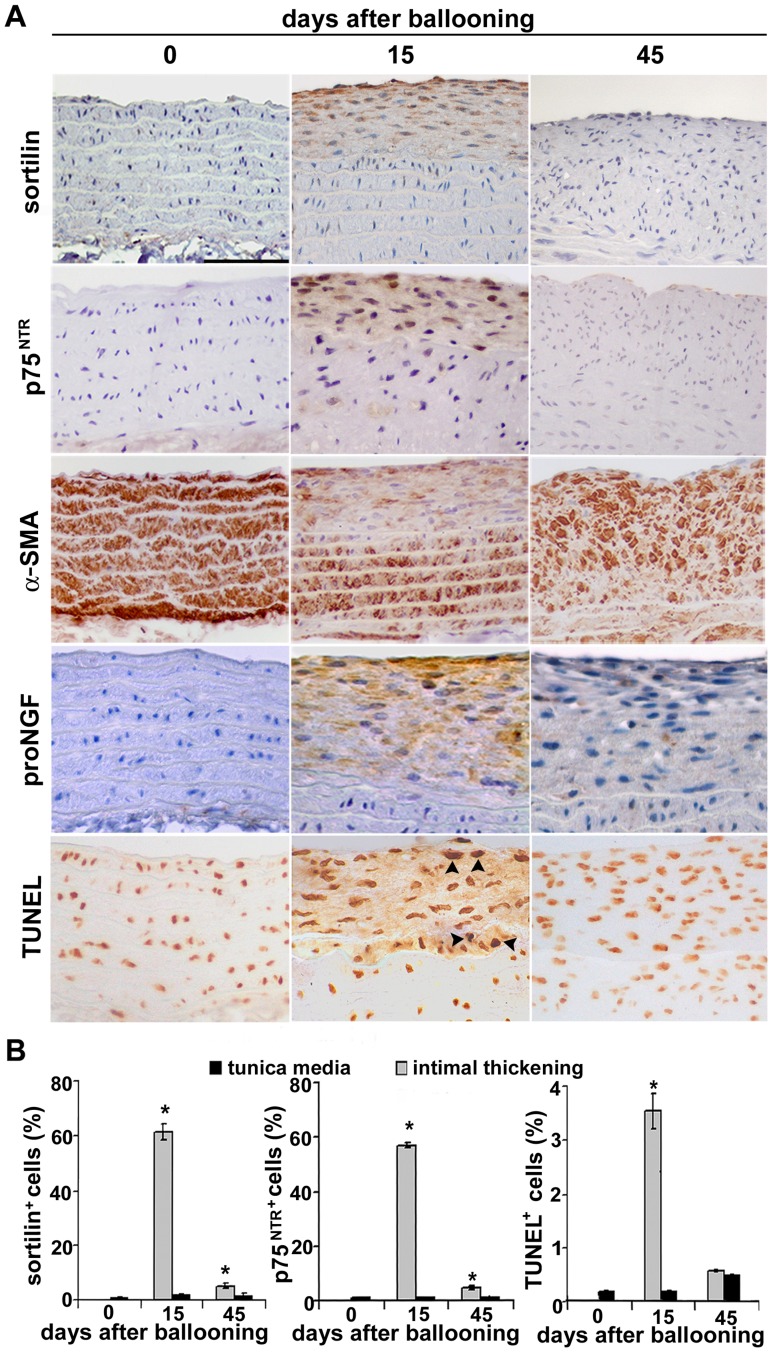
Sortilin expression and apoptosis in normal and post-injury rat aortas. Anti-sortilin and anti-p75^NTR^ immunostainings (A) do not reveal detectable positivity in normal tunica media. Intimal thickening appears markedly sortilin, p75^NTR^ and proNGF positive fifteen days after ballooning, but not after forty-five days; α-SMA immunodetection goes in the opposite direction. TUNEL^+^ cells are evident in the neointima 15 days after ballooning (arrow heads)**.** Bar graphs (B) showing sortilin^+^, p75^NTR+^ and TUNEL^+^ apoptotic intimal cell percentages; *p<0.05. Scale bar = 50 µm.

**Figure 3:**
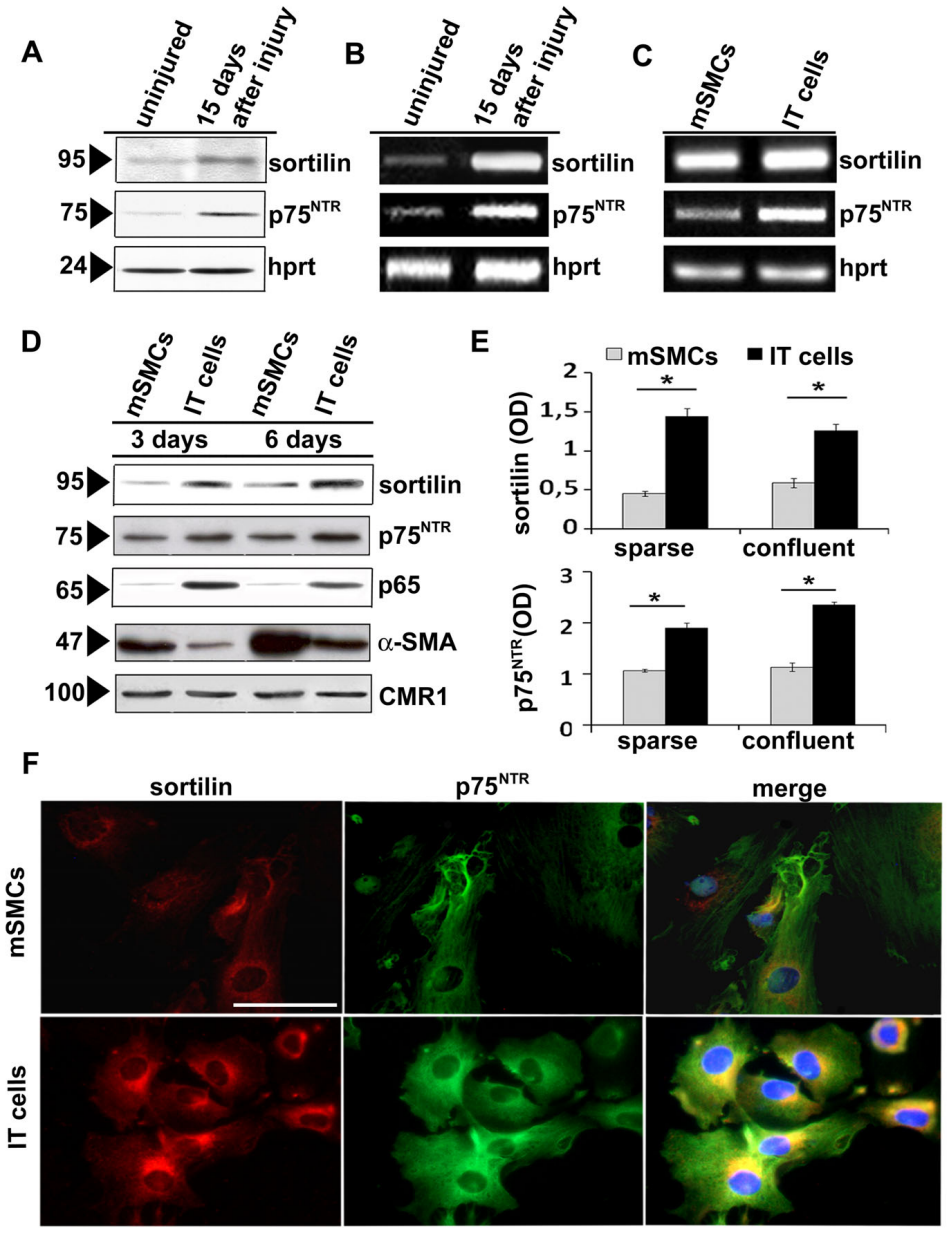
Sortilin expression in rat aortic smooth muscle cell populations. Representative blot analysis of (A) sortilin protein expression and transcript levels (B) in normal rat aortic medial tissue and 15 days after ballooning. Sortilin and p75^NTR^ (C) transcripts accumulate in intimal cells obtained 15 days after injury (IT cells). The latter and normal media SMCs (mSMCs) were harvested after 3 and 6 days in sparse and confluent cultures, respectively. Representative blots and densitometric analysis (D and E) after normalization to CMR1 expression; data are mean ± SEM of three experiments. Sortilin and p75^NTR^ immunofluorescence (F) documents a higher intracellular sortilin and p75^NTR^ level in IT cells compared to mSMCs; right panel: merged images showing the prevalent co-localization of sortilin with p75^NTR^; *p<0.05. Scale bar = 25 µm.

By immunohistochemistry, sortilin was barely detected in normal rat aortas; however, sortilin expression became markedly evident in the intimal thickening fifteen days after ballooning (Fig. 2A and B); also p75^NTR^ and proNGF expression was increased in the neointima, according to that previously reported [17]. Sortilin immunostaining was also observed in neointimal cells three and seven days after ballooning (not shown). Intimal thickening 15 days after injury also displayed low α-SMA expression and increased apoptotic rate compared to underlying and uninjured media (Fig. 2A and B), in accordance with previously reported data [28]. Forty-five days after injury, sortilin and p75^NTR^ expression and the number of apoptotic cells were strongly reduced, while α-SMA was re-expressed**.** Double immunohistochemistry (Fig. S1) showed that 76.5±8% of TUNEL^+^ intimal cells 15 days after injury were also sortilin positive. Western blot and PCR analysis for sortilin and p75^NTR^ confirmed immunohistochemical differences (Fig. 3A and B).

### Increased Sortilin Expression Characterized Intimal Smooth Muscle Cells *in Vitro*

**Figure 4:**
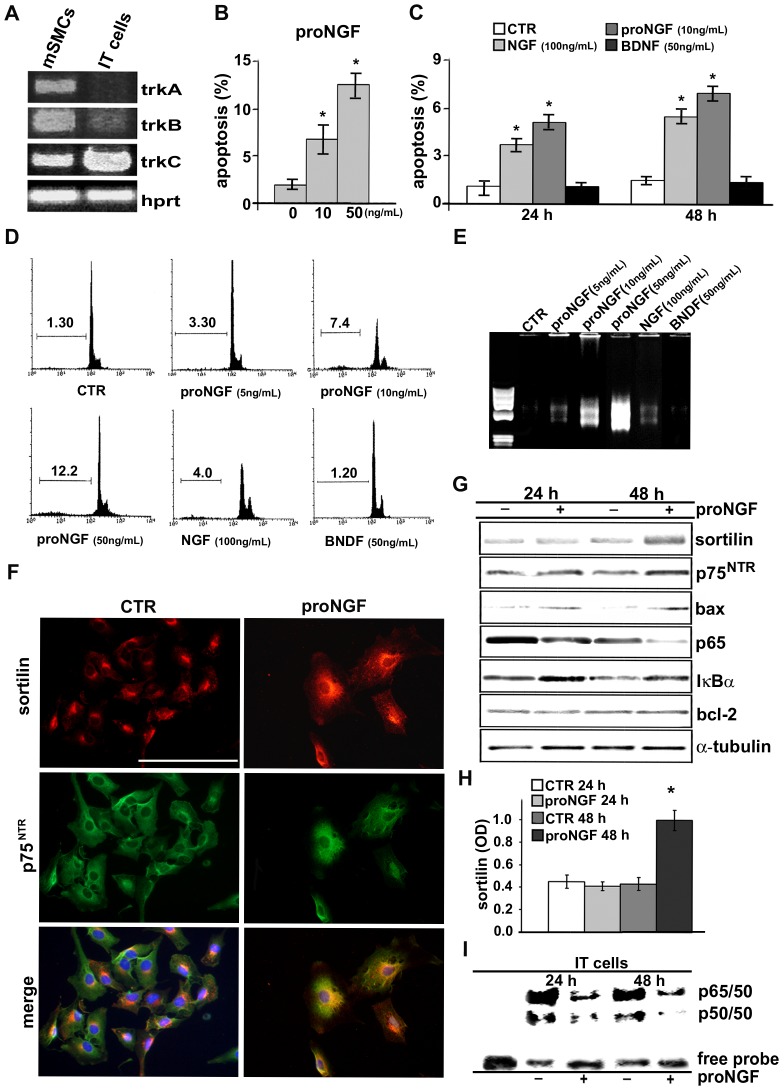
ProNGF is a potent apoptotic inducer of IT cells. IT cells (A) express TrkB and TrkC but not TrkA transcripts, while all Trk receptors are present in mSMCs. ProNGF (B and C) is a potent apoptotic inducer of IT cells and induces a dose-dependent increase of cultured rat aortic IT cell apoptosis as measured by TUNEL in serum-free medium after 24 and 48 hours; flow cytometry (D) of rat aortic intimal cells in sub-G1 (DNA content<2N) calculated as percentages of total events (10,000 cells). Agarose gel under UV light (E) after staining with ethidium bromide showing the ladder production after blunt end linker ligation confirms the dose-dependent and higher proNGF apoptotic DNA fragmentation compared to control IT cells. Representative immunofluorescence (F) of IT cells after 24 h of proNGF (10 ng/mL) treatment shows intracellular distribution of sortilin somehow more evident in the cell membrane compartment, whereas p75^NTR^ localization is almost unchanged**.** Blot analysis (G and H) shows that proNGF (10 ng/mL) induces the increase of sortilin protein content only after 48 h. EMSA analysis (I) in IT cells after 24 h and 48 h of treatment with proNGF shows a significant reduction of NF-κB activity (*upper arrow*, p65/50 heterodimer; *lower arrow*, p50/50 homodimer). Data are reported as mean ± SEM of three independent experiments. *p<0.05. Scale bar = 25 µm.

We investigated sortilin expression in cultured SMC populations from different rat aortic layers. IT cells showed higher sortilin, p75^NTR^, p65 subunit of NF-κB and less α-SMA at both protein and mRNA level compared to mSMCs in both sparse and confluent cultures (*p*<0.05, Fig. 3C–E). In IT cells**,** immunofluorescence (Fig. 3F) documented sortilin distribution in the cytoplasm, likely at level of Golgi complex, and in correspondence of the cell membrane; co-localization of sortilin with p75^NTR^ was clearly present, although somehow less evident in correspondence of the cell membrane. Finally, IT cells expressed transcripts for TrkB and TrkC but not TrkA, whereas mSMCs showed all Trk receptor transcripts (Fig. 4A).

### ProNGF is a Potent Inducer of intimal Smooth Muscle Cell Apoptosis

To investigate the role of sortilin in IT cells, we cultured cells in the presence of the neurotrophins NGF and BDNF and the pro-neurotrophin proNGF. ProNGF induced a marked and dose-dependent increase of IT cell apoptosis, much greater than equimolar or even higher concentration of NGF (Fig. 4B-D), whereas BDNF up to 50ng/mL concentration was ineffective. PCR was also performed on DNA samples extracted from IT cells cultured in the presence of NGF, BDNF and proNGF at different concentrations. Agarose gels showed a dose-dependent and greater apoptotic ladder in proNGF-treated IT cells compared with control (CTR; Fig. 4E). Apoptotic susceptibility of mSMCs was limited and detectable only with very high proNGF and NGF concentration (not shown).

### ProNGF Induces an Early Intracellular Redistribution of Sortilin

Immunofluorescence analysis of IT cells (Fig. 4F) after 24 h of proNGF treatment showed that sortilin distribution somehow more evident in correspondence of the cell membrane than that observed in control IT cells (Fig. 3F), with no significant increase of protein accumulation by blots (Fig. 4F and H), suggesting an early intracytoplasmatic redistribution of sortilin; instead, p75^NTR^ distribution was almost unchanged. Blot analysis documented the successive increase of sortilin protein after 48 h in the presence of proNGF (Fig. 4G and H); instead, sortilin transcripts were already increased after 12 h of proNGF treatment. In parallel, proNGF up-regulated bax (110±20% of control) and IκB-α protein (180±12% of control) after 24 h of treatment and reduced NF-κBp65 subunit after 48 h (43±5.2% of control), whereas bcl-2 appeared unchanged (Fig. 4H). ProNGF also reduced NF-κB activity in IT cells as demonstrated by EMSA (Fig. 4I), supporting a link between proNGF-induced apoptosis and NF-κB activity reduction.

### Sortilin Silencing Prevents ProNGF-induced Apoptosis

**Figure 5:**
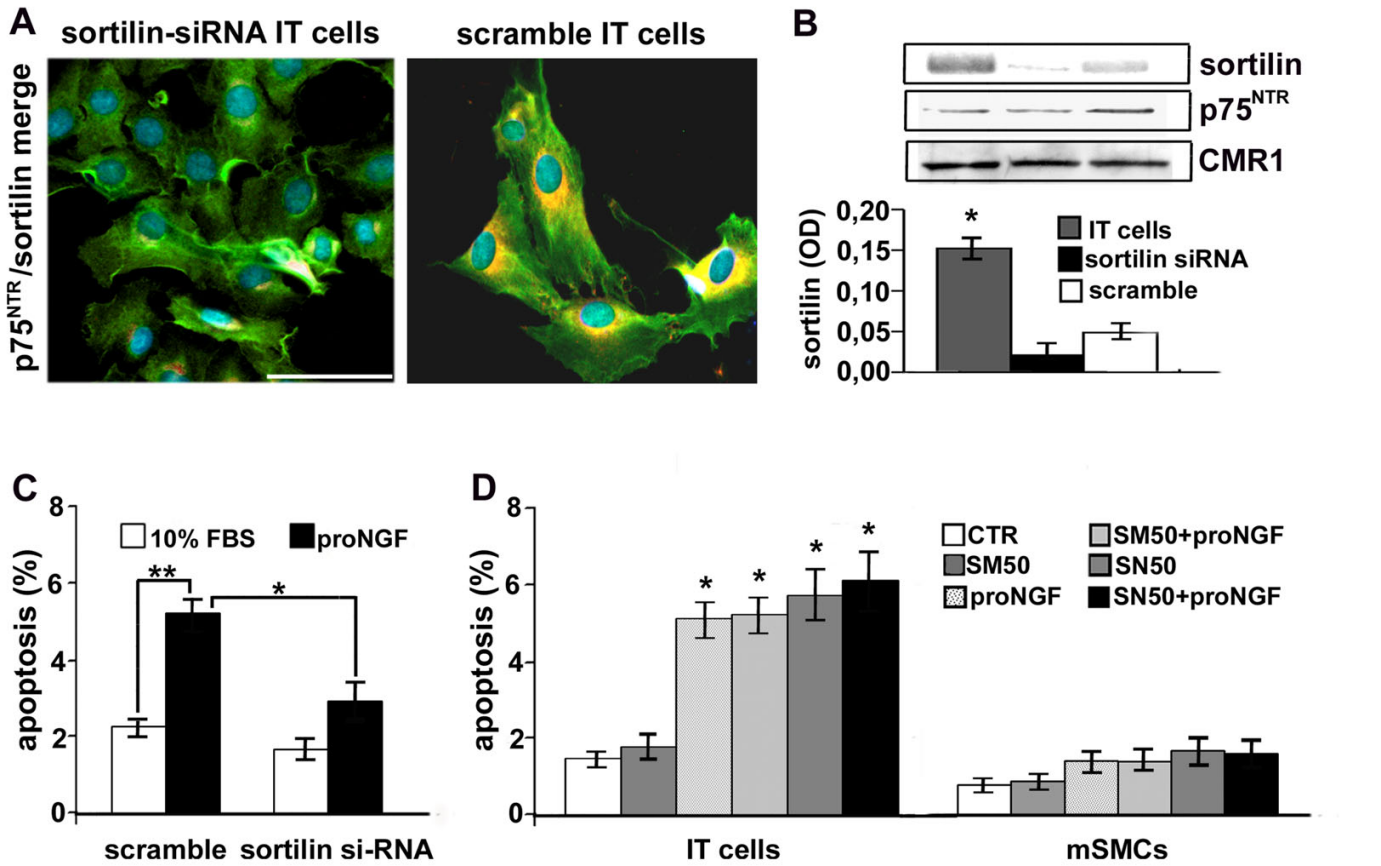
Silencing of sortilin partially prevents proNGF-induced apoptosis of IT cells. Representative immunofluorescence (A) and densitometric analysis of blots (B) showing reduced sortilin expression in sortilin-silenced IT cells. Sortilin silencing (C) reduces 24 h proNGF-induced apoptosis. Graph (D) shows that SN50-induced NF-κB inhibition increases apoptosis of IT cells but not of mSMCs, that doesn’t further increase after successive 24 h of proNGF treatment. Data as mean ± SEM of three independent experiments. *p<0.05. Scale bar = 25 µm.

To better examine its role in proNGF-induced apoptosis, we knocked-down sortilin by using a siRNA. Immunofluorescence (Fig. 5A) and blots (Fig. 5B) confirmed the protein down-regulation in silenced IT cells, whereas scramble transfection was slightly effective. Sortilin silencing did not modify significantly p75^NTR^ level. As reported in Fig. 5C, proNGF-induced apoptosis was lower in sortilin-silenced than in control IT cells (p<0.05), supporting that sortilin expression is needed for proNGF-induced SMC apoptosis.

### ProNGF Doesn’t Further Increase NF-κB Inhibition-induced Apoptosis of IT Cells

We investigated the role of NF-κB during proNGF-induced apoptosis. SN50, an inhibitor of NF-κB nuclear translocation, but not its inactive analogue SM50, increased IT cell apoptosis compared to control (*p*<0.01, Fig. 5D) but not in mSMCs, according to previously reported data [28]. A similar result was obtained with the NF-κB inhibitor PDTC. The addition of proNGF to SN50-treated IT cells did not further increase apoptotic rate after 24 h, supporting that NF-κB inhibition contributes to proNGF-induced intimal SMC apoptosis.

## Discussion

Three major findings are reported in present study. First, increased sortilin expression characterized human atherosclerotic plaques and post-injury rat aortic neointimal thickening. Second, proNGF was proved to be a potent inducer of vascular SMC apoptosis, with higher efficacy than its mature counterpart and other neurotrophins. Third, sortilin expression was required for proNGF-induced SMC apoptosis, likely through a NF-κB-dependent pathway. Cell death plays a prominent role in vascular remodeling and characterizes the progression of human atherosclerosis [32] and experimental post-injury intimal thickening [3], likely counteracting excessive pathological intimal growth. Increased sortilin expression in vascular lesions was parallel to that of p75^NTR^. Sortilin was no more detectable in intimal thickening forty-five days after ballooning, when SMCs display an α-SMA-rich phenotype and apoptosis is markedly reduced [28], in line with a link between the SMC phenotypic heterogeneity and apoptotic susceptibility [11], [33]. Increased sortilin expression was maintained in IT cells *in vitro*; these cells display a dedifferentiated epithelioid phenotype with low α-SMA mRNA and protein expression [11]. Neurotrophin activation classically regulates neuronal survival and differentiation, but also migration, proliferation and apoptosis of other cell types, including SMCs [24], [34]. The existence of a neurotrophin-regulated apoptotic pathway of SMCs was first documented by Bono *et al*., [35]. Successively, the contribution of p75^NTR^ to neurotrophins-mediated apoptosis was also highlighted [17]. Moreover, p75^NTR^ deficiency in knock-out mice reduced apoptosis and favored post-injury intimal thickening [36]. Proneurotrophins and their mature counterparts are variably expressed in embryonic and postnatal vessels [37]. After the documentation that secreted proneurotrophins are capable to influence cell survival by themselves [20] further studies better defined their biological properties. Proneurotrophins demonstrated an important regulatory role, that is distinct from that of mature neurotrophins [20]. Here, we documented that proNGF is a potent inducer of IT cells apoptosis, more than its mature counterpart or others neurotrophins. In neurons, secreted proNGF mediates apoptosis via the binding to a p75^NTR^/sortilin heterodimer [20], [22], suggesting that sortilin function is intimately related to p75^NTR^. Our present results documented that in serum-grown IT cells sortilin co-localizes with p75^NTR^, although in a less evident manner in correspondence of the cell membrane, similarly to other cell types where sortilin localization is also described in the Golgi complex [38]. In SMCs, mature NGF is processed from the 26 kDa precursor proNGF by endoproteolytic cleavage in the Golgi complex, and this process requires the activity of the proportion convertase furin [39]. Moreover, furin determinates the balance between proNGF and NGF in proliferating SMCs, thus impacting on SMC survival [39]. In fact, enhanced NGF expression characterizes proliferating SMCs, and was parallel to the inhibition of proNGF activation. Moreover, sortilin gene and protein levels were reported to be reduced in proliferating compared to quiescent SMCs [39]. Our results support the hypothesis that during the phase of neointimal growth of SMCs in the first days following injury, NGF secretion and TrkA activity prevail, whereas successively, during the remodeling phase characterized from the progressive reduction of proliferation and the increased level of apoptosis, quiescent intimal SMCs produce more sortilin and proNGF and less TrkA in an autocrine loop regulating SMC survival and counteracting excessive intimal hyperplasia following injury [28]. As matter of fact, IT cells did not express significant level of TrkA transcripts, which is generally involved in NGF-mediated survival signals; this finding supports the hypothesis that apoptotic cell death of vascular SMCs derives from the prevalence of p75^NTR^/sortilin-dependent signals on the pro-survival Trk-related signals [20]. Neurotrophin-induced survival and differentiation appears predicted through the binding to Trk receptors [16] whereas the p75^NTR^ binding acts as apoptotic regulator [22]. In particular, NGF has been reported as the preferred ligand of TrkA [20]. It appears more complex to establish the pro-apoptotic effect of proNGF in injured vessels *in vivo*, since p75^NTR^ and Trk are co-expressed [24]. In genetically modified SMCs lacking Trk receptors, neurotrophins interact to p75^NTR^ with a low affinity [40]. Moreover, in human normal vascular SMCs pro-apoptotic activity of NGF is inhibited by TrkA phosphorylation [35] and SMC binding affinity to p75^NTR^ was five-fold greater for proNGF than its mature counterpart [20], revealing the dual opposite function of the activation of these subsets of receptors on SMC survival. In serum-cultured IT cells, sortilin distribution was cytoplasmic and some how less evident in correspondence of the cell membrane as compared to p75^NTR^, in accordance with that previously shown in other cell types [38]. An involvement of sortilin in the intracytoplasmic trafficking of the lysosomial hydrolase acid sphingomyelins has been also suggested [22]. We observed that exposure to proNGF is followed by an early intracellular redistribution of sortilin and a more progressive cell membrane co-localization with p75^NTR^, and only successively the protein content of sortilin increased in IT cells.

Our data highlight the relevance of sortilin expression in the apoptotic machinery of intimal SMCs. The existence of an additional receptor in the neurotrophin-induced apoptotic pathway supported further the fact that not all p75^NTR^-expressing cells respond to proNGF-induced apoptosis [22]. Sortilin was identified as a co-receptor and the molecular switch enabling Trk and p75^NTR^ expressing neurons to respond to proneurotrophins, with a pro-apoptotic rather than pro-survival commitment [22]. The independent role of sortilin in proNGF-induced intimal SMC apoptotic machinery was defined by siRNA experiments. In fact, siRNA sortilin deprivation counteracted proNGF-induced IT cell apoptosis, with no effects on level and distribution of p75^NTR^. Additional studies are needed to verify how SMC phenotypic changes modify the complex balance between spatial and temporal expression of sortilin and p75^NTR^. We documented that the modulation of NF-κB activity accompanies proNGF-induced SMCs apoptosis. The apoptotic effect of NF-κB down-regulation is functionally and phenotypically regulated [13], [27]. Higher NF-κB activity level characterized intimal cells compared to normal media SMCs and its specific inhibition increased SMC apoptosis [28]. NF-κB inhibition-induced IT cell apoptosis did not further increase with successive proNGF treatment. Inappropriate NF-κB activity is reported to modulate apoptosis also in other cell types. In mutant mice with almost complete absence of NF-κBp65 subunit, the absence of NF-κBp65 subunit in mutant mice resulted in an increased number of apoptotic Schwann cells in axotomised distal sciatic nerve segments [41]. It is likely that post-injury dedifferentiation of intimal cells promotes NF-κB-related transcriptional activity and the parallel sortilin and p75^NTR^ overexpression. NF-κB down-regulation is likely to be required to activate proNGF-induced bax-dependent and mitochondrial IT cell apoptosis, and inhibition of IκB-α degradation concurs to this process [28].

In conclusion, we documented the increase of sortilin expression in human atherosclerotic lesions and rat aortic post-injury neointima. ProNGF is a potent apoptotic inducer of IT cells and sortilin plays an important regulatory role during proNGF-induced apoptosis and post-injury vascular remodeling.

## Supporting Information

Figure S1 **Sortilin, α-smooth muscle actin and apoptosis in rat aorta 15 days after ballooning and IT cells** ***in vitro*** **.** TUNEL^+^ cells (A) (black head arrows) are also positive for sortilin immunostaining. Merged image of α-actin (B) (green) and sortiln (red) immunofluorescence. Hoechst staining (C) reveals a condensed nucleus featuring an apoptotic IT cell (white head arrow). Scale bar = 25 µm.
(TIF)
