# Supplementary figures and images for: Sortilin Expression Is Essential for Pro-Nerve Growth Factor-Induced Apoptosis of Rat Vascular Smooth Muscle Cells
Source: PLoS One. 2014 Jan 3;9(1):e84969. doi: 10.1371/journal.pone.0084969 (PMC3880332; doi:10.1371/journal.pone.0084969)

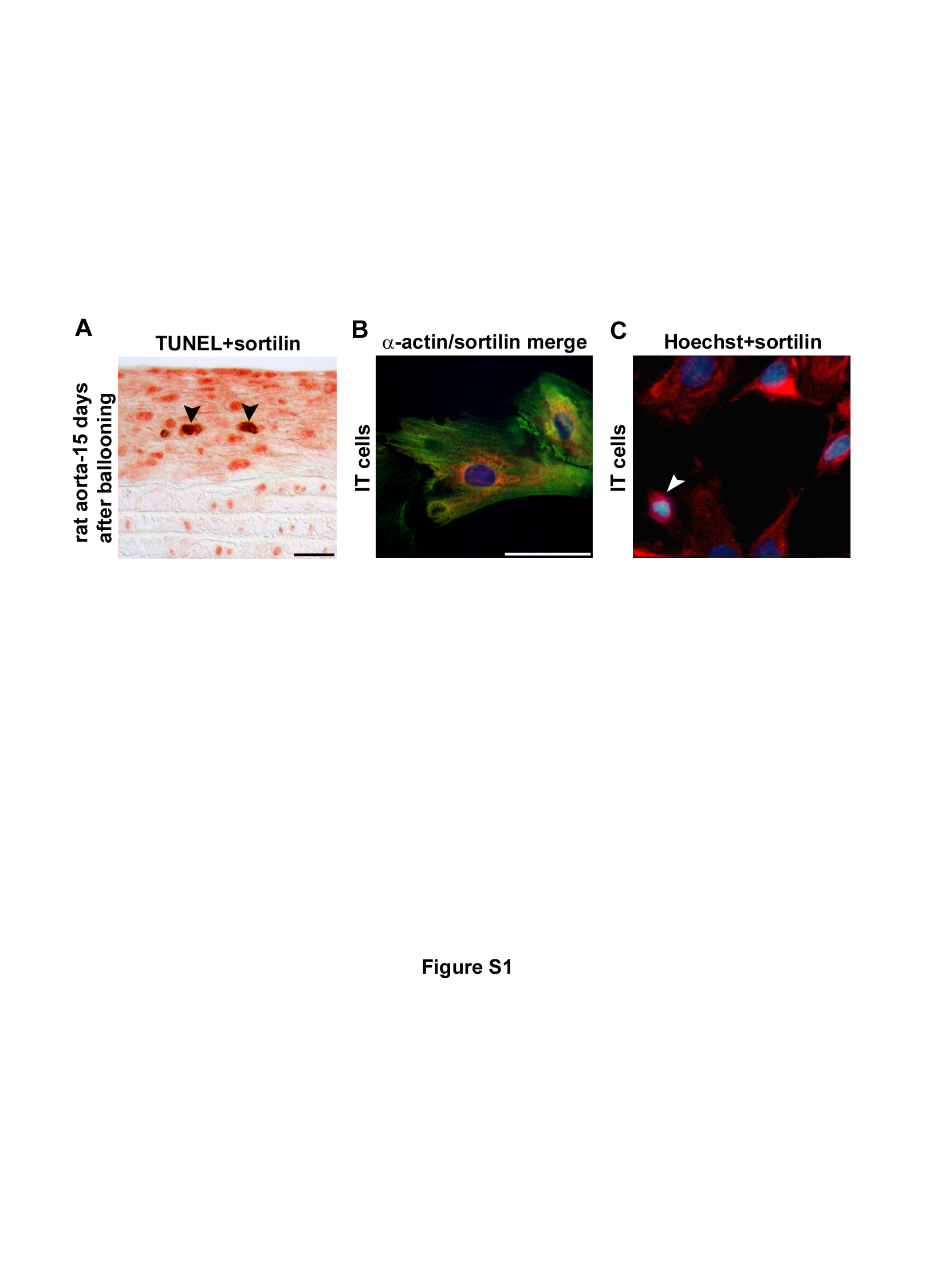

Supplement: Figure S1 — Sortilin, α-smooth muscle actin and apoptosis in rat aorta 15 days after ballooning and IT cells in vitro . TUNEL+ cells (A) (black head arrows) are also positive for sortilin immunostaining. Merged image of α-actin (B) (green) and sortiln (red) immunofluorescence. Hoechst staining (C) reveals a condensed nucleus featuring an apoptotic IT cell (white head arrow). Scale bar = 25 µm. (TIF) [file pone.0084969.s001.tif]
